# Longitudinal study of Asian elephants, *Elephas maximus*, indicates intermittent shedding of elephant endotheliotropic herpesvirus 1 during pregnancy

**DOI:** 10.1136/vetreco-2014-000088

**Published:** 2015-08-04

**Authors:** Laura Bennett, Stephen Dunham, Lisa Yon, Sarah Chapman, Megan Kenaghan, Laura Purdie, Rachael Tarlinton

**Affiliations:** 1 School of Veterinary Medicine and Science, University of Nottingham, Sutton Bonington Campus, Loughborough, LE12 5RD, UK; 2 Twycross Zoo, Burton Road, Atherstone, Warwickshire, CV9 3PX, UK

**Keywords:** Herpesviruses, Elephants, Zoo animals, Virology, Viruses

## Abstract

**Introduction:**

EEHV-1 is a viral infection of elephants that has been associated with a fatal haemorrhagic syndrome in Asian elephants. Previous studies have suggested that pregnant animals may shed more virus than non-pregnant animals.

**Methods:**

This study examined whether pregnancy affected the frequency or magnitude of shedding of elephant endotheliotropic herpesvirus 1 (EEHV1) using Taq man real-time PCR on trunk washes from four female elephants from a UK collection over three time periods between 2011 and 2014. These periods included pregnancies in two animals (period 1 and period 3). Behavioural observations made by keepers were also assessed.

**Results:**

During period 1 there was a high degree of social hierarchical instability which led to a hierarchy change, and was associated with aggressive behaviour. Also during period 1 EEHV-1 shedding was of a higher magnitude and frequency than in the latter two time periods.

**Conclusions:**

These results suggest that there is no clear relationship between shedding and pregnancy, and that behavioural stressors may be related to an increase in EEHV-1 shedding.

## Introduction

Elephant endotheliotropic herpesvirus 1 (EEHV-1) is a betaherpesvirus associated with a fatal haemorrhagic syndrome in juvenile Asian elephants (*Elephas maximus*) (Davison and others 2009). Clinical EEHV disease can be very rapid, beginning with oedema of head and thoracic limbs, lethargy, anorexia and reluctance to drink, and progressing to ulceration and cyanosis of the tongue, and internal organ haemorrhage. Death can occur between one and seven days from onset of clinical signs (Ossent and others 1990, Richman and others 1999, Richman and others 2000, Reid and others 2006, Garner and others 2009, Denk and others 2012). More than 60 cases have been reported worldwide, with a mortality rate as high as 85 per cent (Latimer and others 2011). There is no vaccine or other preventive measures for EEHV infection, but treatment with intensive care and famciclovir has been used, and in some cases, the animal has survived (Schmitt and others 2000). This treatment is expensive: a course of famciclovir can be in excess of £10 000 per animal (US$17 000) (Sharon Redrobe, Personal Communication, 2012 – The cost of famciclovir treatment). EEHV is currently not able to be cultured though qPCR of viral DNA in trunk washes are an established way of detecting EEHV viral shedding (Stanton and others 2010).

It has recently been discovered through studies of zoo animals that many, perhaps most, captive adult Asian elephants intermittently excrete EEHV-1 from oronasal mucosa (Stanton and others 2010, Hardman and others 2011), suggesting that subclinical infection is widespread. Viral DNA shedding can be detected using conjunctival swabs, buccal swabs, trunk swabs and trunk washes (Hardman and others 2011, Stanton and others 2013, Stanton and others 2014). It is not clear why some animals develop fatal haemorrhagic disease and others only have latent or asymptomatic infections. The route of transmission of the virus is also currently unknown though transmission via nasal secretions from the trunk has been suggested (Stanton and others 2010).

Herpesviruses exhibit both latent and lytic (active) phases. In the betaherpesvirus family, latency has been reported in lymphoreticular cells, secretory glands, kidneys and other tissues (Knipe and Howley 2007). Reactivation of human herpesviruses from latency is thought to occur as a result of immunosuppression during stressful physiological states such as occurs in: pregnancy, organ transplant and chemotherapy patients (Sakamoto and others 1982, Greenberg and others 1987, Dahl and others 1999).

Many healthy non-immunosuppressed individuals continuously or sporadically shed various herpesviruses such as Epstein-Barr virus and herpes simplex virus 1 (Junker and others 1991, Kaufman and others 2005, Pierson and others 2005).

Physiological measurement of stress levels in animals can be difficult; however, measurement of cortisol levels is usually accepted as an indicator of stress levels. Cortisol is a product of the hyothalamic–pituitary–adrenal axis, which is activated at times of physical or psychological stress (Foley and others 2001, Möstl and Palme 2002, Millspaugh and others 2007). In many wildlife species, measurement of serum cortisol levels is impractical; obtaining blood from the animal can be stressful, and would interfere with the study as this handling could in itself increase cortisol levels. Faecal glucocorticoid metabolites (breakdown products of cortisol excreted in the faeces) are, therefore, used as a non-invasive measurement of stress in elephants (Millspaugh and others 2007).

Several recent reports from longitudinal studies of EEHV shedding in zoo herds (Stanton and others 2010, Hardman and others 2011) have indicated that EEHV-1 shedding detected by trunk washes may be increased during pregnancy; this trunk secretion could present a possible transmission route to juveniles and neonates. Hardman and others (2011) reported that pregnancy may affect the amount of EEHV virus shed; the pregnant animal in their study had a higher level of virus detected in trunk washes during the third trimester of pregnancy, compared with other stages of the pregnancy, or to the rest of the herd throughout the pregnancy.

The current study is the first longitudinal study of EEHV-1, which has included an examination of shedding during two pregnancies in the same herd. This permitted investigation of the suggestion that pregnancy may increase EEHV-1 shedding frequency and magnitude (Stanton and others 2010, Hardman and others 2011). The current study used qPCR on trunk wash samples to examine EEHV-1 shedding in an all-female closed herd at a European zoological collection. Samples were examined from 2011 to 2014 over three periods: during pregnancies in two animals that occurred at different times (sample period 1 and sample period 3), and during a period when no animals were pregnant (sample period 2). Faecal glucocorticoid levels (for those time periods for which they were available) and behavioural records were also examined to analyse physiological or behavioural indicators of stress that may be linked with EEHV-1 shedding.

## Materials and methods

This study was approved by the School of Veterinary Medicine and Science (University of Nottingham) non Animals Scientific Procedures Act Committee, September 13, 2011.

Samples were collected from four female Asian elephants at a UK zoo. The elephants are referred to as elephant A, B, C and D. Samples were collected during two separate pregnancies, and one period where no animal was pregnant. Both pregnancies were the result of artificial insemination. Elephant hierarchical ranking was reported by the keepers, and was based on observations of dominant and submissive behaviours seen in interactions between the animals over time. A hierarchy change was observed on November 20, 2011 (Table 1).

**TABLE 1:** Elephants included in the study showing keeper-estimated rank, age and detailing pregnancies during three sample periods

Elephant	Hierarchy rank before November 20, 2011	Hierarchy rank after November 20, 2011	Age	Sample period 1 (September 7, 2011 to March 26, 2012) pregnancy status	Sample period 2 (March 27, 2012 to May 22, 2012) pregnancy status	Sample period 3 (May 23, 2012 to March 20, 2014) pregnancy status
A	2nd	3rd	16	−	−	−
B	1st	1st	29	−	−	−
C	4th	4th	30	+	−	−
D	3rd	2nd	19	−	−	+

Pregnancy status of each elephant is also shown: +, pregnant; −, not pregnant

Time period 1 (pregnancy 1) was from September 7, 2011 to March 26, 2012 (samples were taken for seven months during this period). Time period 2 (no animal pregnant) was from March 27, 2012 to May 22, 2012 (two months), and time period 3 (pregnancy 2) was from May 23, 2012 to March 20, 2014 (samples were taken for 11 months during this period). Sampling was not continuous during period 1 and period 2.

One hundred and thirty-seven trunk washes were tested from the four animals over the three sample time periods. Trunk washes were collected by having the elephant draw up 50 ml of saline into its trunk, the trunk was then elevated for 30 seconds, and the elephant then forcefully expelled the saline into a plastic freezer bag. The contents were then transferred into a 60 ml plastic container, and were stored at −80°C until transport and processing of samples could be arranged. All samples were treated alike. Before processing, trunk wash samples were thawed and filtered through gauze to remove sand and feed material, and were then centrifuged at 458 g for 10 minutes. The supernatant was discarded, and cell pellets were stored at −20°C until further processing.

DNA was extracted from trunk wash cell pellets, using a NucleoSpin Tissue Kit (MACHEREY NAGEL), as per manufacturer's instructions. To avoid cross-contamination, all work was conducted in a class II laminar flow hood, and a separate plastic opener was used for each 1.5 ml microcentrifuge tube.

Quantitative PCR analysis was conducted using a modified version of the protocol described by Stanton and others (2010). However, alternatively sourced primers and probes and equipment brands were used, and the quencher dye on the probe was modified from MGB-NFQ to Eclipse.

EEHV viral shedding was normalised against interferon-γ (IFN-γ) using a standard curve; the qPCR cycle threshold value was converted into a copy number (the number of EEHV and IFN copies within each sample), and the number of EEHV copies was divided by the number of IFN-γ copies in each sample to obtain a ratio.

Freidman tests with Dunn's multiple comparison test as a post-hoc test were performed in GraphPad Prism V.5.04 (GraphPad Software) for comparative viral load (a) between animals and (b) median viral loads during each sampling period.

Faecal samples were opportunistically collected at irregular intervals from all animals. An aliquot of faeces was collected from the centre of the faecal bolus, and the material was manually broken apart and transferred into a 60 ml universal container. Faecal samples were stored at −20°C until analysis. Faecal glucocorticoid and progesterone analyses were performed using the protocol described by Watson and others (2013).

Elephant keepers maintained records of the animal's behaviour; events recorded included submissive behaviour, aggressive behaviour, management changes, foot care and medical observations. Records were stored on the MedARKS database. Foot care events occurred at the same incidence throughout the study, and no major illnesses or injuries were sustained during the study period.

## Results

**FIG 1:**
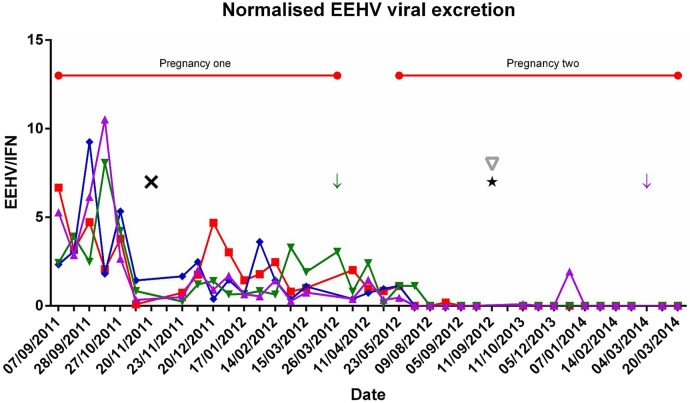
Elephant endotheliotropic herpesvirus (EEHV) viral shedding in four female elephants. EEHV was normalised against interferon-γ (IFN-γ). Red squares=elephant A, blue diamonds=elephant B, green inverted triangles=elephant C, purple triangles=elephant D. Green arrow=elephant C gave birth, purple arrow=elephant D gave birth, black star=switched to protected contact, black X=change in hierarchy, grey inverted triangle=change in keeping staff and red horizontal bar=periods of pregnancy. The y axis is the amount of EEHV normalised against IFN-γ. EEHV was normalised against IFN-γ. EEHV copy number was divided by IFN-γ copy number to give a ratio. On the x axis is the date; note that dates were not continuous, as sampling periods were irregular

During ‘sample period 1’ (pregnancy 1), all samples from all animals (61) tested positive for the presence of EEHV, with normalised viral loads between 0.1 and 10.5. During ‘sample period 2’ (non-pregnant period), all samples (12) also tested positive for EEHV-1 though at a lower magnitude of normalised viral load (below 3.1). During ‘sample period 3’ (pregnancy 2), EEHV-1 was detected in 8 out of 63 samples (12.69 per cent) though these samples had an even lower normalised viral load of below 2.0 (Fig 1).

From August 2011 until October 2011, before the change in hierarchy, elephants B, C and D showed elevated levels of EEHV. There was a drop in EEHV shedding in all animals in November 2011. This was then followed by a period of consistent shedding in all animals from December 2011 until June 2012. From August 2012 until March 2014, EEHV-1 was detected in 81 out of a total of 137 samples (59.56 per cent) (Fig 1).

The median magnitude viral load for each elephant was: elephant A, 1.28; elephant B, 1.17; elephant C, 1.18 and elephant D, 1.21. Elephant D had the highest frequency of positive samples at 61.7 per cent (22 out of 34). All samples were positive in sample periods 1 (pregnancy 1) and 2 (non-pregnant period). In sample period 3 (pregnancy 2), elephant D had the highest frequency of positive samples at 18.7 per cent (3 out of 16) (Table 2).

**TABLE 2:** Percentage of positive samples for each elephant

Sample period	Elephant A	Elephant B	Elephant C	Elephant D
1	100% (15/15)	100% (15/15)	100% (16/16)	100% (15/15)
2	100% (3/3)	100% (3/3)	100% (3/3)	100% (3/3)
3	13.3% (2/15)	6.3% (1/16)	15.5% (2/16)	18.7% (3/16)
Total	60.6% (20/33)	55.8% (19/34)	60% (21/35)	61.7% (22/34)

No significant differences were apparent in normalised viral load between animals. However, median viral loads during time period 1 and 3 were significantly different (Freidman test P value 0.0046, Dunn’s multiple comparison test time period 1 v time period 3 P<0.05).

**FIG 2:**
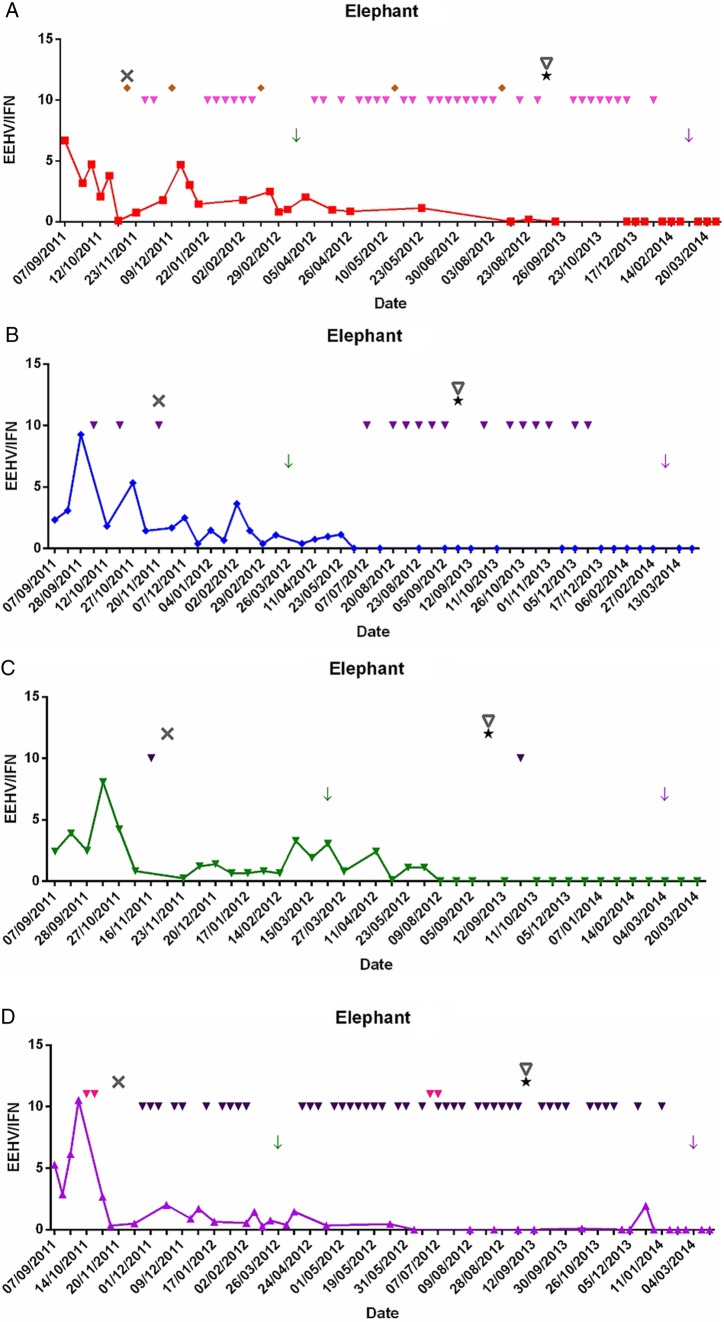
EEHV viral shedding and incidents of aggression in four female elephants. Red squares=elephant A, blue diamonds=elephant B, green inverted triangles=elephant C, purple triangles=elephant D, green arrow=elephant C gave birth, purple arrow=elephant D gave birth, black star=switched to protected contact, black X=change in hierarchy, grey inverted triangle=change in keeping staff, orange diamond=injury/reopened injury from aggression, purple inverted triangle=incidents of aggression/tension (aggressor) and pink inverted triangle=incidents of aggression/tension (victim). Incidents involving more than one elephant were classed as 1 occasion, aggressive behaviour may also only involve a single animal where an animal exhibited aggressive behaviour, but other animals were not involved. EEHV, elephant endotheliotropic herpesvirus; IFN, interferon

A total of 50 incidents of aggression were recorded by keepers. During sample period 1, there were a total of 14 incidents. Over the five months of this period, this equated to an incident rate of 2.8 per month. During this time period, elephant A suffered three injuries from aggressive behaviour from elephant D, and overall, injuries were most common during this sampling period. This is also the time during which the first pregnancy occurred. During sample period 2, 13 incidents of aggression were observed, and one injury was sustained by elephant A. The rate of incidents during this time equated to 6.5 incidents per month. During the third sample period, the second pregnancy occurred. There were a total of 23 incidents in this sampling period; the incident rate was 2.9 per month, with one incident causing an injury to elephant A (Fig 2).

Faecal glucocorticoids were monitored for short periods, which only partially overlapped with trunk wash sampling. No patterns of variation in glucocorticoid levels versus normalised viral load were evident (data not shown).

A number of samples were Sanger sequenced (Source BioScience), and the presence of EEHV-1 DNA was confirmed.

## Discussion

In this study, EEHV was detected in four out of four elephants though results suggest that pregnancy did not result in increased EEHV-1 shedding; indeed, median normalised load during the second pregnancy was significantly lower than during the first, which was not the case for the difference between normalised viral loads for either pregnancy and the non-pregnant period in this study. Freeze thawing of the trunk wash samples may have resulted in some cell lysis and a reduced sensitivity of the present assay; however, all samples were positive for IFN DNA, indicating that cellular DNA was present, and have been presented as a ratio of viral to cellular DNA to compensate for variation in input DNA. During the first pregnancy, all four elephants shed EEHV-1 consistently. During the second pregnancy, shedding was quite sporadic. Furthermore, during the period when no animal in the group was pregnant, shedding was continuous. This is in contrast to herpesvirus reactivation during human pregnancy, which has been reported for human herpesvirus 6 (Dahl and others 1999) and human herpesvirus 4 (Sakamoto and others 1982).

The study herd was closely monitored, and data were available for a number of other factors that might be linked to stress in the animals (and EEHV-1 shedding). The faecal glucocorticoid results are difficult to interpret without previously defined baseline levels, but no clear patterns emerged between faecal glucocorticoid levels and EEHV-1 shedding. However, because there were a limited number of samples used for hormone measurements during the sampling periods for EEHV-1 testing, these results must be interpreted with caution.

Keeper reports of behaviour were used to identify aggressive behaviours throughout the study period. Dominance-related aggression relating to hierarchy change has been observed in many other species, and incidents of aggression may occur for several days until a hierarchy is established (Houpt 2011). Studies in other species have also indicated that corticosterone measures are more strongly linked with intensity of aggression than with frequency of incidents (Marsteller and others 1980).

In this study, the highest level of EEHV shedding occurred during sample period 1 (pregnancy 1). This is the period during which keepers reported that a hierarchy change occurred on November 20, 2011. Elephant A also sustained an injury on this date due to aggressive behaviour. Analysis of the behavioural records showed that a large number of incidents involved elephant D as the aggressor and elephant A as the victim. This was not unexpected as these were the two elephants that the keepers reported had switched in their hierarchical ranking. Although there were not as many aggressive incidents during sample period 1 compared with other sample periods, the intensity appears to be greater as indicated by the higher number of injuries or reopened injuries that resulted during this period. This period of hierarchical instability could have led to higher levels of stress in the herd, and suggests that EEHV shedding may be linked to behavioural stress in these animals.

Elephant management practices at the facility also changed during this study. Until September 11, 2012 (during sample period 2), the herd was managed using a ‘free contact’ approach (where keepers have direct physical contact with the animals). After this date, the management style changed to ‘protected contact’ (where there is always a physical barrier between the keeper and the elephant (Association of Zoos and Aquariums 2011). There was no clear connection between shedding and management methods, as frequency of viral shedding and magnitude of viral load were already decreasing by this time, and decreased to negligible levels after these management changes.

The results from this study suggest that that there is no clear relationship between shedding of EEHV-1 in captive Asian elephants and pregnancy. However, behavioural stressors may be related to an increase in EEHV-1 shedding. It is also possible that EEHV shedding is affected by other as yet unidentified factors, or that the animals may have been newly infected when the first sample set was taken. Further longitudinal studies on greater numbers of elephants, targeted monitoring of glucocorticoid levels, detailed behavioural observations and more detailed data on any behavioural indications of stress would add further support to the findings suggested by this study.
